# How Ethical Issues Raised by Human–Robot Interaction can Impact the Intention to use the Robot?

**DOI:** 10.1007/s12369-021-00857-8

**Published:** 2022-01-13

**Authors:** Reza Etemad-Sajadi, Antonin Soussan, Théo Schöpfer

**Affiliations:** grid.508733.a EHL, HES-SO, University of Applied Sciences Western Switzerland, Lausanne, Switzerland

**Keywords:** Human-robot interaction, Ethical issues, Trust and safety, Social cues, Autonomy, Responsibility, Privacy and data protection

## Abstract

The goal of this research is to focus on the ethical issues linked to the interaction between humans and robots in a service delivery context. Through this user study, we want to see how ethics influence user’s intention to use a robot in a frontline service context. We want to observe the importance of each ethical attribute on user’s intention to use the robot in the future. To achieve this goal, we incorporated a video that showed Pepper, the robot, in action. Then respondents had to answer questions about their perception of robots based on the video. Based on a final sample of 341 respondents, we used structural equation modeling (SEM) to test our hypotheses. The results show that the most important ethical issue is the *Replacement and its implications for labor*. When we look at the impact of the ethical issues on the intention to use, we discovered that the variables impacting the most are *Social cues, Trust and Safety*.

## Introduction

The service sector has always been a laboratory for innovations, as it is an inflection point between productivity and personalization. In this matter, technologies such as AI, clouding, and data banks have been implemented to revolutionize the future of the industry. Robotics, of course, is also a newcomer in the service sector. Robots used in this field are called “service robots”. They are defined as “system-based autonomous and adaptable interfaces that interact, communicate and deliver service to an organization’s customers” [68].

Whenever a new technology emerges, it tends to induce fear and a lack of trust. The interactions can be complicated as some people do not understand the technology and can be biased towards it. Ethical considerations play an essential role in the interaction between the consumer and technology, as society creates an ensemble of implicit and explicit rules to protect itself. Privacy, security, liability, dehumanization, and unemployment are part of the main concerns users may have [18]. Nevertheless, robots are no exception to the rule and tend to exacerbate these fears. As personifications of technology, they have been the object of fantasy for many years. Besides, because service robots, compared to other robots, work directly with clients of a company they have an immediate impact on the reputation of the company. Robots, and especially service robots, have proven to be useful in “dull, dirty, or dangerous” tasks [44], p. 4), but they can take different forms: functional and social. For our research, we want to identify ethical issues that have an impact on the interactions between humans and service robots. Are robots being used to save money by replacing humans? How is our personal data used during (and after) an interaction with a robot? To what extent is the behavior of robots unpredictable? All of these questions can have an impact on the intention to use service robots. The safety of human beings is questioned since a mistake made by a robot could lead to dangerous situations. People are also afraid that robots will replace them, which would pose economic and human unemployment problems. This would create dependency on robots. Psychological risks are also predicted, such as problems of attachment, fears, or the confusion between artificial and real [66]. Some existing regulations could be applied to the notion of safety with robots. An example would be the Code of Ethics of the Association for Computing Machinery (ACM), which states that: “When designing or implementing systems, computing professionals must attempt to ensure that the products of their efforts will be used in socially responsible ways, will meet social needs, and will avoid harmful effects to health and welfare” [47], p. 354).

The originality of this research stems from the fact that potential users will have to define the importance of each ethical attribute on their overall intention to use service robot in the future. This empirical study will therefore be centered on participants’ assessment of certain ethical items. This method should allow us to uncover which ethical items are important for the customer’s intention to use a service robot. Moreover, we focus our ethical issues applied to service robots in order to have more concrete ethical guidelines for this precise technology. However, the aim of this paper is not to uncover novel ethical issues, but to build on the ethical dimensions discussed in the existing literature to test the customers’ intention to use service robots. Finally, this article will not be about all types of robots (such as military, medical, or industrial robots), but will focus solely on social robots used in a service delivery context.

### Service Robots

Service robots are robots specialized in acting in a service delivery context. Their unique feature is that they interact with human customers socially. They can also be defined as social service robots [68], as they can create some degree of automated social presence. They make the customer feel that they are with another social entity [63]. The literature states that there are three main attributes that should be taken into consideration in a service context: representation, anthropomorphism, and task orientation [68]. The representation can be split into two different categories: virtual (e.g., Alexa by Amazon or Google assistant) or physical (e.g., Pepper or SARA Singapore's Automated Responsive Assistant), which is the case for our research. The embodiments can be anthropomorphic according to the human-like characteristics, but behavior can also contribute to the perception of anthropomorphism which can be split into two distinct categories: humanoid (e.g., NAO or Pepper manufactured by Softbank robotics) or non-humanoid (e.g., Roomba the vacuum-cleaner robot). Lastly, the task orientation can differ between basic preprogrammed tasks (e.g., Roomba the cleaning robot), more cognitive-analytical tasks (e.g., image analysis software assistant for medical diagnosis), or socio-emotional tasks (e.g., SARA Singapore’s Automated Responsive Assistant) [68]. The same authors listed different characteristics that distinguish service robots and frontline employees on a micro (service training, learning, and customer experience), meso (market level), and macro dimension (societal level).

### Ethics and Robots

“The emergence of the robotics industry is developing in much the same way that the computer business did 30 years ago” [21]. As developed by Calo [7], robotics has a specific set of essential qualities and it generates distinct ethical and legal issues. When diving into the literature about robots, we can observe that most researchers support this statement. On the other hand, some concerns about the impact of robotics have emerged within society, as documented by numerous researchers, mainly because this technology could be unpredictable and potentially dangerous. For most technologies, the first concern is the safety of the product. Robotics is no exception to this rule. As robots are coded by human engineers and often consist of millions of lines of code, errors can occur. While these mistakes may not harm people in ‘more basic’ technological applications, they can be more problematic in the context of robotics. A great example is when, in 2010, the U.S. military lost control of a drone for more than thirty minutes. The drone violated a restricted airspace by flying towards Washington D.C. before operators managed to re-establish communication with it [6]. Another concern is hacking, meaning that ill-intentioned individuals could take control of the robot. Furthermore, it is still unclear who should be responsible in case the robot makes an error. Holding robots responsible for their actions, especially if they are social robots, could lead to a better acceptance of them. However, affective responses towards robots differ from those towards human agents, implying that the responsibility is not perceived as valuable [62]. Lin et al. [45] adds that a natural way to minimize the risks that a robot could pose would be by programming them to obey laws and codes of ethics. Nevertheless, it remains an open question as to which ethical code or punishment should apply.

The social presence of robots is another gray area that will impact laws and regulations as well. KASPAR and IROMEC, for example, are specifically designed to be social mediators for children with special needs (e.g., Autism Spectrum Disorders) [42]. Lin et al. [45] explored the degree of companionship a robot should have: could it be a companion like a human or a pet? Could it be used as a drinking partner or a sexual partner? Or should a robot just be a “slave”?

Privacy is another issue to consider in this context. Indeed, research about privacy has proven the importance of this factor in a human–human context (medical records, lawyers, and clients). Research was then generalized to human–machine interactions: data privacy [54]. Furthermore, it is now known that "data is the new oil" [4]. Companies sell data, and they are considered the world's most valuable resource. More recently, the European Union has addressed this concept with the General Data Protection Regulation (GDPR), which became effective on May 25, 2018. It is the first and most important regulation concerning data privacy. The regulation focuses on several key requirements: consent of subjects for data processing, anonymizing collected data to protect privacy, providing data breach notifications, safely handling the transfer of data across borders and requiring certain companies to appoint a data protection officer to oversee GDPR compliance [11]. If companies do not respect this regulation, they can pay fines up to 4% of their total global turnover or EUR 20 million.

Robots use lots of data to function at their full potential. In the context of service delivery, this could translate into the preferences of a customer or facial recognition for personalizing the customer experience. A robot needs to have this information to understand their interaction partners and thus behave intelligently [30]. They are, therefore, subject to data privacy and related legislation.

As with any industrial or technological revolution, one of the biggest concerns is job losses. Some experts argue that every job that will disappear is being replaced by a new one [8]. A standard response to the job-loss concern is that humans will be able to use more energy where they can make more impact [45]. In other words, robots could improve working conditions (by doing menial tasks) and human productivity. Empathy, communication, creativity, and flexibility seem to be a competitive advantage against robots [12].

Trust is one of the major element in any social relation. People can become dependent on technology [15], which is why it has become an important issue in the development of new technologies. Technology has become such an important social actor that the characteristics of the relation are similar to human–human relationships. A sense of security, credibility, reliability, loyalty, and accuracy then applies to technology as well [50].

As robots become increasingly ubiquitous, ethical questions have become a central preoccupation. Experts have tried to identify critical ethical attributes and remedy them through ethical charters. The first one known was written by the science-fiction novelist Isaac Asimov in 1942. The European Robotics Research Network (EURON) decided to create, in 2005, *The Roboethic Roadmap* to provide a systematic assessment of ethical issues involved in the area of robotics R&D. Finally, in 2007 in South Korea, a panel of experts met to adopt a robotic ethical charter. The idea was to create an ethical guideline to define the roles and functions of robots in the future [53].

Through this study, we want to explore the way ethics influence user's intention to use a service robot. We want to observe the importance of each ethical attribute on user’s intention to use the service robot in the future. In the next section, we will share our research model and hypotheses.

## Research Model and Hypotheses

**Fig. 1:**
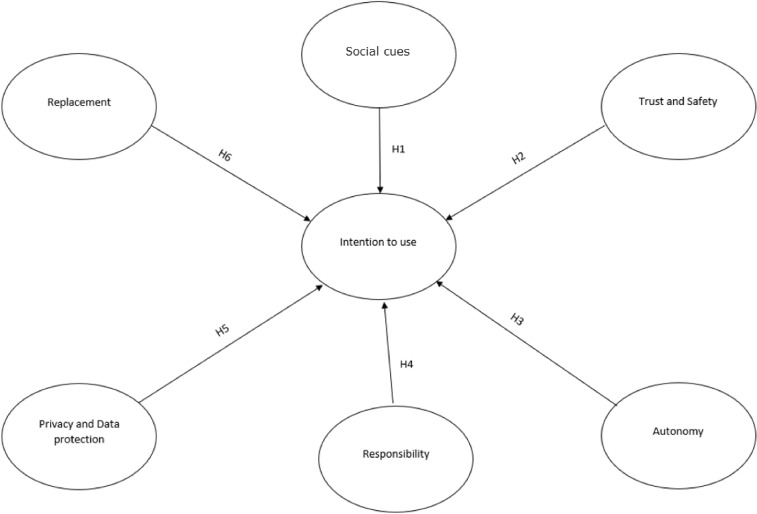
Research model

In this research, we will try to identify factors related to ethical issues that also impact the intention to use the robot in the future in a service delivery context (e.g., restaurant, hotel, hospital, etc.). Figure 1 shows our research model, and our hypotheses will be defined hereafter.

We have seen that service robots’ appearance and behavior can closely resemble those of humans [68]. Research has proven that too close a resemblance to a human might affect the acceptance of a robot [14]. By human behaviors, we intend cases in which a robot acts as if it were human in a service delivery context, regardless of the shape of the robot (e.g., we might imagine a frontline service robot shaped as dog with human behaviors). Therefore, we will take this point into consideration in assessing the customers’ intention to use the robot. A robot, in addition to delivering a service, needs to be a social actor (any person who undertakes social action). It can be translated by the fact that robots can be pleasant conversational partners or can even play a social role in a team. As defined by Darling [10], a “social robot” is a physically embodied, autonomous agent that communicates with humans through social cues, learning adaptively and mimicking human social states. van Doorn et al. [63] theorized that a service robot’s Automated Social Presence (i.e., giving the impression that there is someone else present with us) might have an impact on the customer experience. However, social presence is not the only characteristic which can make robots human-like. Emotions are important, and, according to de Kervenoael et al. [12], empathy could play a crucial role in Human–Robot Interaction (HRI). The authors showed that empathy has a significant and positive impact on the intention to use a social robot. Acting like a human is not only being able to display emotions, but also having facial expressions. In a study where participants played a game (Akinator) with a robot, researchers demonstrated that if the robot mirrors the facial expressions of the person with whom it is interacting, the intention to use the robot increases compared to when the robot does not display any facial expressions [25]. As Wirtz et al. [68] highlight, for human–robot relations to be successful, robots need to act like humans do. Indeed, they need to follow social norms and display the right emotions at the right time. In the context of service delivery robots this dimension can be directly and/or indirectly linked to ethics. Indeed, through the interviews also that we did before this study (with companies using robots, associations linked to AI & ethics, Robot companies providers, etc.), we found that the social/human cues can be an antecedent for measuring the intention to use and for defining ethical issues. When we ask for example the following question to the users: “I perceive robots as social actors”, one can claim that this question can be evaluated/interpreted through an ethical point of view. For this reason, we decided to integrate this dimension to our model. Therefore, we hypothesize that the more a robot acts like a human, the higher the user’s intention to use it will be.

### H1

The more a robot is expected to act like a human (social cues), the higher the user’s intention to use it.

The next hypothesis focuses on the notion of trust. Lee & See [41] summarized several definition of trust and the way it can be measured according to each context. The focus was on the dimension of trust in automation, beliefs, attitudes, intentions, and behaviour. As mentioned in their article, people respond socially to technology and therefore trust influences reliance on automation. A simple definition of trust consistent with these considerations is the attitude that an agent will help achieve an individual’s goals in a situation characterized by uncertainty and vulnerability [41]. The level of trust in a service robot can impact the user intention to use it in the future. Etemad-Sajadi & Sturman [16] also found that trust has a significant impact on the intention to use a service robot. Indeed, for a product/service to be trusted, it needs to be safe. The issues could be mostly with their software design [45] and lead to non-negligible problems. The safety issues robots could pose are related to physical, psychological and emotional damages. As for the physical safety of users, Salvini et al. [54], p. 456) notice that “As a matter of fact, predictability, namely, the possibility to tell in advance a machine behaviour, which is a fundamental criteria for determining the safety of industrial robots, is not applicable to a service robot, due to its ability to generate autonomous behaviour in response to changing environments”. Vasic & Billard [64] share the same opinion: they observed that mobile robots are increasingly present in workplaces where they do not have an assigned functioning space, as would be the case for industrial robots. In this context, as the authors argue, robots rely on their sensor to avoid physical contact with humans, which can sometimes be fallible. Robots are also vulnerable to cyberattacks, which can create dangerous situations for users. Denning et al. [13] cite multiple possible attacks that people could possibly be subjected to with robots at home, such as an attacker eavesdropping on conversations being had in the home. Finally, researchers have shown that people can be emotionally attached to their robots (Roomba™ the vacuum cleaner robot in this case), to the point of caring and worrying about them [59]. In the context of robots in elderly care, Sparrow & Sparrow [55] argue that, for now, robots are “not capable of real friendship, love, or concern” (p. 154). They concluded that technology is only deceiving users into thinking they are being cared for. The question of emotional harm can therefore be asked: could there be dependence on robots which could harm users emotionally [52]? According to these points, we hypothesize that the more the robot is safe and trustworthy, the higher the user’s intention to use it.

### H2

The more a robot is safe and trustworthy, the higher the user’s intention to use it.

It is still unclear to which extent a robot should be kept under supervision, controlled, or act on its own. This study aims to consider this point. Beer et al. [2] define autonomy as “the extent to which a system can carry out its own processes and operations without external control” (p. 77). In other words, autonomy is the degree to which a robot can do its tasks without a human’s input. The concept also refers to the ability of a robot to adapt to its environment and is crucial to study HRI [60]. We hypothesize that the higher the perception of a robot’s autonomy, the higher the user’s intention to use it will be.

### H3

The more a robot is expected to act autonomously, the higher the user’s intention to use it.

When a problem occurs among humans, responsibility is an inevitable legal and psychological process. In the case of robotics, it is still unclear who should be responsible, as laws and regulations are still emerging in this area, and the philosophical debate is still ongoing (cf., among others: [28, 38, 48, 56]. In an empirical study, Furlough et al. [20] found that when an error occurs, people tend to blame humans first, then robots and finally the environment. In their study, when the robot was described to the participant as autonomous, it received more blame than when it was described as non-autonomous, although it still received less blame than humans. These results were corroborated by a study conducted by Leo & Huh [43], who found that, in case of service failures, humans tend to attribute less blame to robots than humans. With these results in mind, it appears important for a company (i.e., humans,) to take responsibility for errors, even when those mistakes are made by a robot. Therefore, we hypothesize that the more a company seems to be responsible for their robot’s acts, the more customers will intend to use the robot.

### H4

The more a company, through its use of a robot, seems to be responsible for the robot’s acts, the higher the user’s intention to use it.

Privacy and data protection have been a central subject for a few years now, especially with the adoption of GDPR and some scandals like Cambridge Analytics during the American presidential campaign. It is an important concern and thus a point to take into consideration in our research. Robots are equipped with sensors such as cameras and microphones in order to navigate their environment and interact with people. However, this also means that they are capable of collecting information and data, which can be seen as a risk for the privacy of customers [68]. Moreover, customers do not always appear to understand the kind of data collected by a robot [40]. Similarly, users may be biased into thinking that their privacy is more protected than it really is. Indeed, preliminary results by Tonkin et al. [61] showed that embodied systems (i.e., having a more anthropomorphic physical form) tend to decrease customer privacy concerns compared to non-embodied ones, which means that embodied robots might collect more data than a non-embodied one. There are laws in place that regulate data collection and protect individuals, such as the General Data Protection Regulation (GDPR) in the European Union [34]. Nevertheless, it is important to assess how privacy and data protection impacts the user’s intention to use a robot. An empirical study led by Vitale et al. [67] found that transparent systems (i.e., a system openly communicating the privacy policies it uses) result in a better user experience (out of the 5 UX variables: more attractive, more dependable, more stimulating and more novel) than systems that are not transparent. Consequently, we hypothesize that the more a robot is seen as a threat to privacy and data protection, the lower the user’s intention to use it will be.

### H5

When the robot is seen as a threat to privacy and data protection, it impacts negatively the user’s intention to use it.

In every technological revolution, people tend to be afraid of the revolution’s impact on jobs and unemployment. As robots tend to be more precise, they can process a large amount of data and act rapidly. They will, therefore, replace certain jobs. On the other hand, they can be a great partner in improving the productivity and efficiency of human workers or in completing unpleasant or menial tasks [8]. As Salvini et al. [51] highlighted, the price of a robot has decreased while the cost of human labor has increased, and the authors argue that the same will surely happen with social robots in the near future. This fact can naturally raise concerns about the place of humans in the workforce. Moreover, the International Federation of Robotics (who are of the opinion that humans will remain competitive and that automation will create new jobs (IFR, [37]) stated that robots can improve a company’s productivity for certain tasks which they execute more efficiently than humans (IFR [36]). According to a survey analysis conducted by Morikawa [46], about 30% of human workers fear being replaced by either a robot or AI, which is substantial but far from constituting a majority of workers. Looking at the data of a European citizens’ survey, Granulo et al. [27] also reported that citizens tend to be scared of being replaced in their jobs by robots. As Salvini et al. (2010, p. 456) argue, “One of the strongest social motivations for not accepting a robot, perhaps above and beyond safety and aesthetic considerations, is related to the widespread feeling that robots can take over jobs that are traditionally the domain of humans”. In the present research paper, we hypothesize that when robots are seen as a potential threat to their employment, the user’s intention to use a robot decreases.

### H6

When a robot is seen as a threat to human jobs, it decreases the user’s intention to use it.

## Methodology

### Measures, Sampling and Data collection procedures

Response options for each item have been ranked from 1 (*strongly disagree*) to 7 (*strongly agree*). Table 1 summarizes the constructs and the items.

**Table 1:** Questionnaire items

Constructs	Items	Adapted from
Social cues	I want robots in a service delivery context to be human-like I want robots in a service delivery context to act like humans I perceive robots as social actors (any person who undertakes social action) in a service delivery context	Beer et al. [1], Gefen et al. [23]
Trust and Safety	I perceive robots as safe in a service delivery context I think that robots in a service delivery context are vulnerable to hackers (i) I would hesitate to use robots in a service delivery context for fear of making errors that will harm me (i)	Stahl and Coeckelbergh [57], Gefen et al. [23]
Autonomy	I think a robot in a service delivery context should deliver limited tasks (i) I think a robot in a service delivery context should be able to act on its own	Stahl and Coeckelbergh [57], European Union's Convention on Roboethics (2010)
Responsibility	I think the law, and subsequent punishment, should apply to robots in a service delivery context The company is responsible for the robot’s actions in case a client is wrongly informed by the robot	Lin et al. [45], Stahl and Coeckelbergh [57],
Privacy and Data protection	I should be informed of how robots will use information about me I don’t mind giving personal information to a robot in a service delivery context (ex: name, age, food preferences for informative robots, nature of my illness for a healthcare robot, etc.) (i)	Graeff and Harmon [26], Stahl and Coeckelbergh [57]
Replacement	I think robots in a service delivery context will contribute to unemployment I think robots in a service delivery context can improve the working conditions of human coworkers (i)	Lin et al. [45]
Intention to use	Assuming I could have access to a robot in a service delivery context, I would use it Assuming I could have access to a robot in a service delivery context, I would prefer to use it instead of a human Overall, I was impressed by robots in a service delivery context I would recommend to the people surrounding me to interact with a robot in a service delivery context	Venkatesh [65], Hellier et al. [31]

**Fig. 2:**
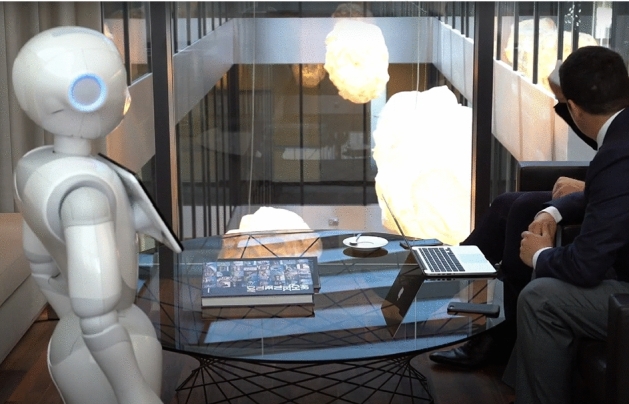
The pepper robot in action

It was important for us that everybody would start the survey with some basic knowledge of and experience with robots in a service delivery context. To achieve this goal, we incorporated a video that showed Pepper, the robot, in action on the campus of an international business school in Switzerland. The Fig. 2 shows Pepper in action, going to a table of potential users and having a conversation. The robot was controlled with the Avatar Remote Control application on a tablet (developed by our partners from Heigvd). Questions asked by the users were very diverse, ranging from “How is the weather?” to precise questions about the school.

Then respondents had to answer questions about their perception of robots based on the video. The estimated duration for answering to our survey was 10 min. The survey was taken by a panel of very different people, as everyone in the future could potentially interact with this kind of robot. To do so, we decided to share the survey via four main platforms: Prolific, WhatsApp, Facebook, and LinkedIn. We also compared the results according to their sources in order to identify if we would have significant differences between the clusters. No significant differences were observed. In the end, we obtained 341 responses, out of which 57.1% of respondents were female, and 42.9% were male. The average age of people taking the survey was 33 years old and the median was 29 years.

### Data Analysis Method

Structural equation modeling (SEM) was adopted to test the hypotheses due to the fact that the model contains several latent variables. SmartPLS 3.0 was used for the analysis. We employed a bootstrapping method (500 sub-samples) to test the significant level of regression path coefficients. We used the blindfolding approach (cross-validated communality and redundancy). The quality of each structural equation was measured by the cv-redundancy index (i.e., Stone-Geisser Q^2^). Q^2^ measures the extent to which observed values are reconstructed by the model and its parameter estimates [9]. The technique represents a synthesis of function fitting and cross-validation [32]. If its value is around 0.35, it means that there is a high predictive relevance [29, 32]. In this model, the independent variables are therefore good predictors of intention to use, as Q^2^ is equal to 0.327.

## Results

### Reliability and Validity of Measures

Table 2 shows that all latent variables have a composite reliability higher than 0.7, confirming that the scale reliabilities have adequate and stable measurement properties. Convergent and discriminant validity are components of a larger measurement concept known as construct validity [58]. Convergent validity is shown when each measurement item is strongly correlated with its construct. It is usually satisfied by retaining variables whose loadings are high, indicating that they share sufficient variance with their related construct. Discriminant validity is confirmed when each measurement item is weakly correlated with all other constructs except with the one with which it is theoretically associated [24]. With PLS, convergent and discriminant validities are confirmed if each construct Average Variance Extracted (AVE) is larger than its correlation with other constructs. Moreover, each item should load more highly on its assigned construct than on the other constructs [22, 58]. Table 2 shows the intercorrelation of the research constructs. The diagonal of this matrix represents the square root of the average variance extracted. For adequate discriminant validity, the diagonal elements should be significantly larger than the correlation of the specific construct with any of the other constructs and should be at least 0.5 [17]. In our case, one can claim that discriminant validity is confirmed and sufficient to support the model.

**Table 2:** Reliability and discriminant validity

Constructs	Composite reliability	1	2	3	4	5	6	7
1. Autonomy	0.70	0.75 ^a^						
2. Social cues	0.83	0.32	0.79					
3. Intention to use	0.89	0.36	0.52	0.82				
4. Privacy and data protection	1	− 0.35	− 0.37	− 0.49	1			
5. Replacement	1	− 0.11	− 0.01	− 0.10	0.17	1		
6. Responsibility	1	0.08	0.16	0.17	− 0.04	0.13	1	
7. Trust and safety	0.89	0.325	0.267	0.57	− 0.44	− 0.20	0.06	0.89

a =  > Diagonal: (Average variance extracted)^1/2^ = (Σλ_i_^2^/n)^1/2^

### Results and Discussion

**Fig. 3:**
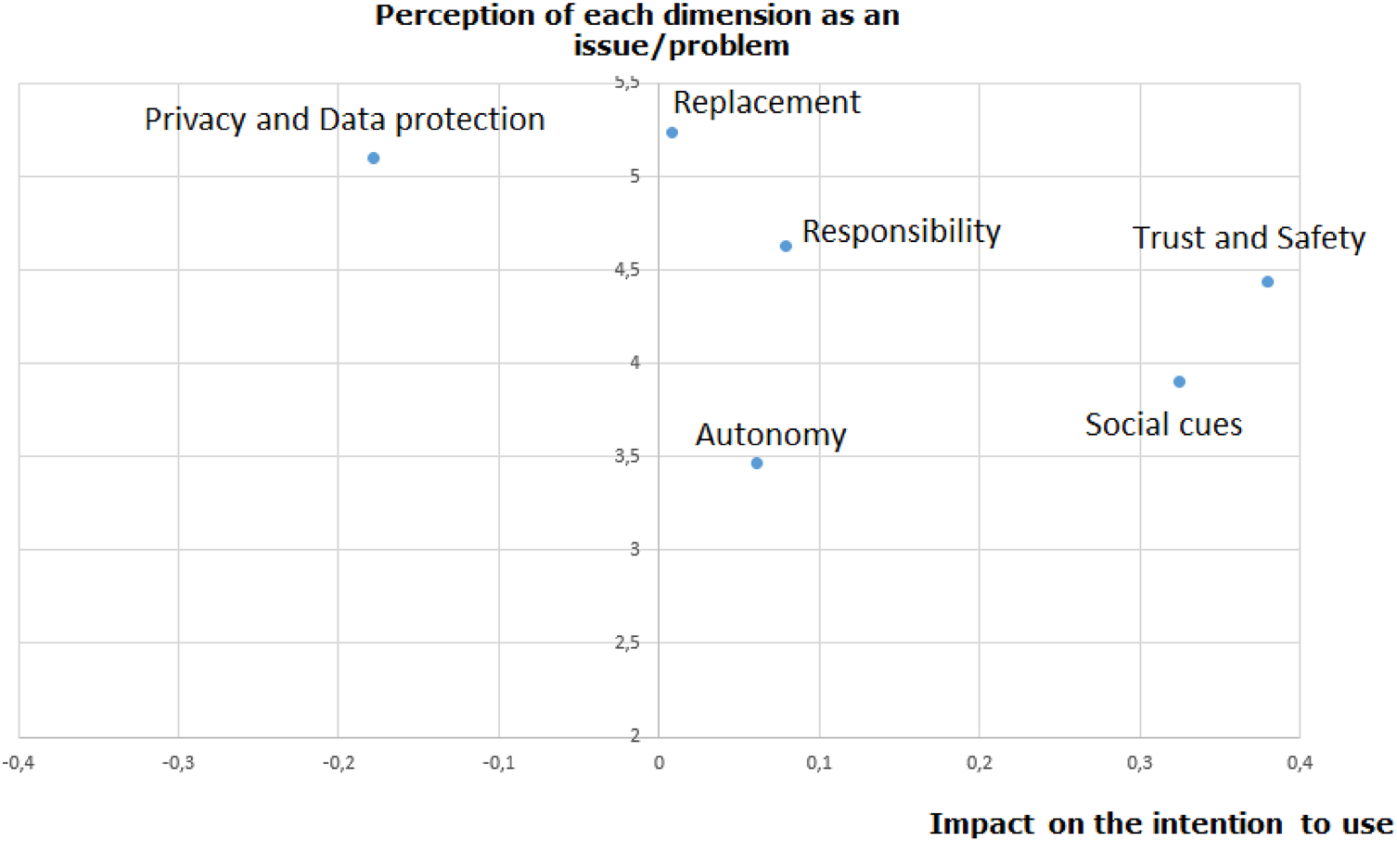
Perception of each dimension and the impact of the intention to use

**Fig. 4:**
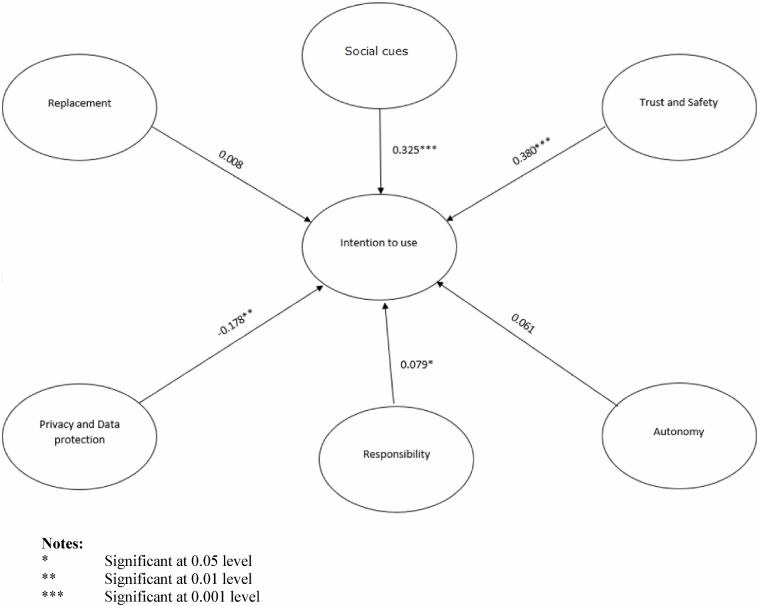
Results of the PLS analysis

Figures 3 and 4 show our results. On Fig. 3, the vertical axis represents the mean for each latent variable and the horizontal axis represents the impact of the independents variables on the “intention to use”. Focusing first on the vertical axis, we observed that the most important ethical issue is the “replacement and its implications for labor” (i.e., I think robots in a service delivery context will cut employment) with an average of 5.25 on a Likert scale from 1 to 7 (SD = 1.35). The second dimension is the “privacy and data protection” (i.e., I mind giving personal information to a robot in a service delivery context) with an average of 5.09 (SD = 1.36). The third most worrying dimension is “responsibility” (i.e., I think the law, and subsequent punishment, should apply to robots in a service delivery context) with an average of 4.62 (SD = 1.87). The value of means for “trust and safety”, “social cues”, and “autonomy” are respectively 4.43 (SD = 1.48), 3.9 (SD = 1.44), and 3.46 (SD = 1.23).

When we measure the impact of each ethical issue on the intention to use the robot in the future, we observe that “trust and safety” (i.e., I perceive robots as safe in a service delivery context) is the most impacting variable on the decision whether to use the robot. Therefore, our hypothesis *H2* (γ = 0.380) is accepted. The second variable impacting the intention to use a service delivery robot is “social cues” (i.e., I perceive robots as social actors in a service delivery context), and this confirms our hypothesis *H1* (γ = 0.325). The third variable impacting negatively the intention to use the robot is “privacy and data protection”. This result confirms hypothesis *H5* (γ =  − 0.178). The fourth variable impacting the intention to use is the “responsibility”. Indeed, *H4* is barely accepted (γ = 0.079). As far as “autonomy” (i.e., “I think that a robot in a service delivery context should be able to act on its own”) is concerned, one can claim that the impact is not relevant to the intention to use and that the perception of this dimension as an ethical problem is also not very high compared to the other ethical issues. This is also the case for the variable named “replacement”. Therefore, *H3* and *H6* are rejected. Our model explains 50.7% of the intention to use and therefore one can claim that our ethical issues are good predictors of the intention to use the robot Table 3.

**Table 3:** Summary of the hypotheses

Constructs	Hypotheses	Status
Social cues	H1: The more a robot is expected to act like a human (social cues), the higher the user’s intention to use it	Confirmed
Trust and Safety	H2: The more a robot is safe and trustworthy, the higher the user’s intention to use it	Confirmed
Autonomy	H3: The more a robot is expected to act autonomously, the higher the user’s intention to use it	Rejected
Responsibility	H4: The more a company, through its use of a robot, seems to be responsible for the robot’s acts, the higher the user’s intention to use it	Confirmed
Privacy and data protection	H5: When the robot is seen as a threat to privacy and data protection, it impacts negatively the user’s intention to use it	Confirmed
Replacement	H6: When a robot is seen as a threat to human jobs, it decreases the user’s intention to use it	Rejected

## Conclusion

This study has shed light on the extent to which ethical issues influence the intention to use robots. Four out of six items were found to have a significant effect on the intention to use a service robot: social cues, trust/safety, responsibility, and privacy/data protection. The two hypotheses which were rejected, namely autonomy and human replacement, do not play a role in the intention of a customer to use a robot. However, it does not mean that these variables do not constitute important ethical considerations. When we look at the fear of human replacement, the perception of this dimension is very high meaning that people are afraid of being replaced. That said, this variable is not directly impacting the intention to use the robot. It can be interesting to see in the future the indirect impact of these dimensions (autonomy and human replacement) on the intention to use.

### Managerial Implications

Robots in a service delivery context are still not very common. Researchers, engineers and service delivery professionals should not forget the ethical questions linked to the ambition to test and use robots in the future. As an example, Battistuzzi et al. [33] developed a research ethics training module in the context of culture-aware robots and environmental sensor systems for elderly support. This training can help researchers conduct experiments in an ethically appropriate manner.

Our research can help service delivery professionals and engineers to understand how they can make robots more desirable by also considering ethical issues. Robots in a service delivery context will become increasingly omnipresent, and they can genuinely enhance service quality. While a good return on investment for hotels [71], they must understand how robots can be used and programmed to maximize guests’ intention to use. We found that the sentiment of trust and safety is the factor that impacts the intention to use the robot the most. Moreover, we saw that robots are expected to display social cues. In order to optimize the use of robots, we advise companies to heed the following ethical concerns. Below you will find the 6 dimensions:

1. *Social Cues* According to our findings, the more a robot displays social cues, the higher the user’s intention to use it will be. Therefore, robots should deliver a service that is as human-like as possible and, thus, include social features. However, a service robot should not hinder or replace human-to-human interactions [5]. It is important to guarantee this aspect when a company want to use robots in a service delivery context.
2. *Trust and Safety* The extent to which a robot is deemed safe and trustworthy is important to the user’s intention to use the technology. Although it can be argued that designers and producers are responsible for creating robots that are safe for users [47], companies using a service robot must always guarantee this major dimension.
3. *Autonomy* Even though, in our case, this variable did not have an influence on the user’s intention to use a robot, the idea of being able to restrict a robot’s autonomy can be found in ethical charters. Therefore, we argue that a company using a service robot should always be able to regulate a robot’s autonomy, especially in cases when the consequences of the robot’s actions cannot be totally controlled.
4. *Responsibility* A robot’s responsibility for its actions is important for the user’s intention to use the technology. Therefore, companies using a service robot should pay attention to this point and clearly define, before the deployment of their robot, who is responsible for the robot’s actions [44], pp. 8–10,[47]. Moreover, to ensure liability, a robot’s actions and decisions must always be traceable [35].
5. *Privacy and Data Protection* Privacy and data protection play a big role in the intention to use a robot. First, a company using a service robot should always respect its customers’ right to privacy [49]. As transparency (i.e., disclosure about what, how and why is collected) leads to a better user experience [67], we advise companies (and their robots) to be transparent regarding the collection and use of their customers’ data [35]. Secondly, companies using service robots should ensure that they protect their customer’s data by encrypting and safekeeping them. Third, companies should always make sure that the robot’s data collection follows official guidelines and local laws [49]. Finally, as mentioned by Fosch-Villaronga & Millard [19], several legal and regulatory questions have to be considered by the integration of physical robotic systems with cloud-based services.
6. *Human Workers Replacement* Although this variable was not found to be important in our model, best practices in relation to the subject can be established. A company should incorporate its employees in the choices and decisions related to the service robot, such as the choice of the robot, or the decisions related to the definition of its tasks [3]. In case a robot should take a worker’s job, the firm should retrain its employee for a new occupation [3].

### Limitations

This research paper has several limitations. First, as demonstrated by Beer et al. [1], socio-demographic factors are a key determinant of the acceptance and the intention to use a robot. Our research did not evenly represent ages or cultures/nationalities. Second, we decided to introduce our survey with a 1-min video in order to align the different respondents. This method may have altered some answers to the survey. Moreover, the video showed the robot Pepper in a service delivery context. Therefore, the results might be only generalizable to robots similar to Pepper in shape (i.e., humanoid robots which are still far from resembling closely to a human). Indeed, there are many different types of service robots which have not been tested and therefore we have to be careful in the generalization of our findings. Third, we have to be careful on the generalization of our findings due to the external validity and our scenario-based study compared to a real interaction. Moreover, there is a “wow” effect when you interact for a first time with a service robot and after several interactions, the experience can become usual. Fourth, this exploratory research sometimes highlighted the weakness of some items. In addition to this point, the low reliability has forced us to delete, in certain instances, an important number of items. That said, in the end, we have four accepted and two rejected hypotheses, and more than 50% of our dependent variable is explained.

### Suggestions for Future Research

For future research, we advise extending the geographic scope to minimize the influence of socio-demographic factors. Another way to work with this problem would be to use two determined perimeters with different socio-demographic factors (e.g., Switzerland and Japan). As this technology is still new and some people might be afraid to use it, in the future, a constructed experimentation and survey could be applied regularly (every five to ten years) to monitor the evolution of ethical issues and the intention to use robots. Moreover, the data of this research was collected before the COVID-19 outbreak. In the context of the pandemic, people have to protect themselves and others with measures such as social distancing. Consequently, human-to-human interactions have been more complicated than before. However, an interesting aspect is that robots can help in the current situation, and different solutions have been considered or developed (e.g., development of a telerobot for healthcare applications: [69], global review of robots during the COVID-19 pandemic with a focus on the tourism and hospitality field: [70]. With this in mind, people might get more used to interacting with robots, and the variables influencing their intention to use, or the quality of service delivered by a robot might also change. As Kim et al. [39] showed, during health crises such as the COVID-19 pandemic, people tend to prefer a robot-based service than one delivered by humans, whereas they prefer the opposite (or a mixed-model) in ‘normal’ times. Therefore, it would be interesting to replicate the present study in the mid-term future, to see whether the ethical items selected are still relevant to the intention to use a service robot, or if other ethical dimensions arise.

## Data Availability

The datasets analysed during the current study are available from the corresponding author on reasonable request.
